# SARS-CoV-2 Virion Stabilization by Zn Binding

**DOI:** 10.3389/fmolb.2020.00222

**Published:** 2020-09-17

**Authors:** Silvia Morante, Giovanni La Penna, Giancarlo Rossi, Francesco Stellato

**Affiliations:** ^1^Dipartimento di Fisica, Università di Roma “Tor Vergata”, Rome, Italy; ^2^INFN, Sezione di Roma Tor Vergata, Rome, Italy; ^3^CNR, Insitute of Chemistry of Organometallic Compounds, Firenze, Italy; ^4^Centro Fermi - Museo Storico della Fisica e Centro Studi e Ricerche “Enrico Fermi”, Rome, Italy

**Keywords:** SARS-CoV-2, Zn binding, molecular dynamics computer simulation, zinc finger, tetherin/Bst2, orf7a and orf8 proteins

## Abstract

Zinc plays a crucial role in the process of virion maturation inside the host cell. The accessory Cys-rich proteins expressed in SARS-CoV-2 by genes ORF7a and ORF8 are likely involved in zinc binding and in interactions with cellular antigens activated by extensive disulfide bonds. In this report we provide a proof of concept for the feasibility of a structural study of orf7a and orf8 proteins. A conceivable hypothesis is that lack of cellular zinc, or substitution thereof, might lead to a significant slowing down of viral maturation.

## 1. Introduction

The SARS-CoV-2 open reading frames ORF7a and ORF8 code for virion non-structural, called accessory (Coffin et al., 1997), proteins of yet unknown function (orf7a and orf8, respectively, hereafter). The protein orf7a is common to all SARS-CoV type coronaviruses and highly conserved (Wu A. et al., 2020), while orf8 is remarkably different from proteins coded by genes ORF8 and ORF8b of human SARS-CoV (Xu et al., 2020).

The orf7a protein of SARS-CoV-1 has been shown to interact with several host proteins (Vasilenko et al., 2010). An analogous situation occurs in the case of the very similar SARS-CoV-2 orf7a protein (Gordon et al., 2020). The most supported hypothesis proposed for orf7a protein function is the interference with virion budding tethering (Bonifacino and Glick, 2004) operated by cellular antigens (Taylor et al., 2015). Indeed, orf7a is expressed in the host cell to inhibit the intracellular (at endoplasmic membrane) process of virion immobilization before and after virion vesiculation. On the basis of structural similarities (Swiecki et al., 2013) we argue that also protein orf8 can be involved in the same inhibition process, strengthening the inhibition of virion immobilization. This conjecture is in agreement with Gordon et al. (2020) where it is suggested that orf8 plays a role in vesicle trafficking and in endoplasmic reticulum protein quality control, thus favoring the reconfiguration of ER/Golgi trafficking during coronavirus infection.

Virion tethering is mainly due to proteins of the tetherin family, also known as bone marrow stromal antigen 2 (BST2) or cluster of differentiation 317 (CD317). BST2 is expressed in many cells in the interferon-dependent antiviral response pathway. The mechanism of tethering involves tetherin protein dimerization via formation of extended disulfide bonds within the coiled coil region (Le Tortorec et al., 2011). This step is known to be strongly influenced by divalent cations involved in Cys binding. Among these ions, the most available in cells is Zn^2+^.

A timely computational search for therapeutic targets of SARS-CoV-2 found the orf7a-BST2 complex as a potential target to be addressed with structural studies (Wu C. et al., 2020).

The orf7a and orf8 protein sequences (both 121 amino acids long) hint at a high Zn binding propensity, as they display 6 Cys/3 His and 7 Cys/4 His side-chains, respectively, with motifs that are able to bind Zn, thus forming multiple zinc finger (ZF) domains. The relevance of Zn in the viral replication process has been widely investigated (Chaturvedi and Shrivastava, 2005; Chasapis, 2018) and, indeed, two ZF domains have been discovered in the nucleocapsid protein of HIV-1 (Ncp7) (Morellet et al., 1998; Guo et al., 2000). Two such similar Zn domains have been also identified (Kirchdoerfer and Ward, 2019) along the highly conserved, within the SARS-CoV family (Xu et al., 2020), nsp12 polymerase.

## 2. Method and Results

The pivotal role of Zn in SARS-CoV replication has been demonstrated by inhibition of RNA polymerase activity both *in vitro* and in cell culture (te Velthuis et al., 2010).

**Figure 1:**
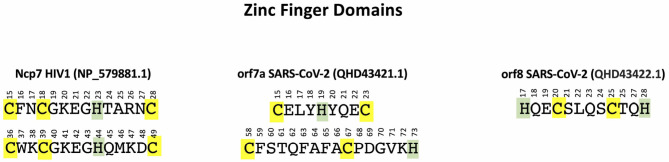
ZF domains in NP_579881 (Ncp7 of HIV-1) **(left)**, QHD43421.1 (orf7a of SARS-CoV-2) **(center)**, and QHD43422.1 (orf8 of SARS-CoV-2) **(right)**. Cys are highlighted in yellow and His in green.

Comparing with the well assessed ZF domains of Ncp7 CX_2_CX_4_HX_4_C (located from aa 15 to aa 28 and from aa 36 to aa 49), one identifies the similar CX_3_HX_3_C and CX_8_CX_5_H motifs in orf7a (located from aa 15 to aa 23 and from aa 56 to aa 73, respectively) and the HX_2_CX_4_CX_2_H motif in orf8 (located from aa 17 to aa 28). The patterns of these motifs are shown in Figure 1.

UNIPROT (https://www.uniprot.org/help/zn_fing) reports a large variety of ZF domains among which also the Cys-rich patterns of orf7a and orf8 can be accommodated.

From a structural point of view it is not difficult to come up with a plausible protein structure capable of hosting a Zn^2+^ ion. Focusing on orf7a as an example, one can proceed to construct such structures by first building an atomistic model of the protein in a random coil configuration. After simulating self-avoiding random walks (La Penna et al., 2004; Furlan et al., 2008) of the chain in vacuum by randomly changing the dihedral angles (not involving H atoms) of all residues (except Φ in Pro), for a total of 445 dihedral angles, we proceeded by monitoring along the trajectory the distance *d* between pairs of Sγ (Cys)-Sγ (Cys) atoms, *d*(CC), and Sγ (Cys)-Nϵ(His) atoms, *d*(CH). All His side-chains are neutral and protonated at Nδ. We collected a trajectory of 400 random configurations, with consecutive configurations separated by 500 attempted torsional moves. Acceptance ratio was about 0.5. Two chain configurations where three among all of the possible *d*(CC) and *d*(CH) distances are smaller than 1.2 nm were selected. This condition was found to be satisfied by the triplets Cys(15)…His(19)…Cys(23) in one case and Cys(58)…Cys(67)…His(73) in a second case. Zn^2+^ ions were then inserted at the geometric center of each of the two selected triplets of atoms. A cationic dummy atom model for Zn (Pang, 2001) and the PARM14 Amber force-field (Maier et al., 2015) for the protein were used. We took a distance cut-off for non-bonding interactions of 0.5 nm, with Coulomb interactions shifted and damped to achieve local neutralization (Wolf et al., 1999). Starting from the selected configurations, we relaxed the systems by classical molecular dynamics (MD). At the beginning, harmonic forces with *k* = 10 kcal/mol/Å^2^ were added to bring the distances between Zn and the three atoms [Sγ (Cys(15)), Nϵ(His(19)), Sγ (Cys(23))] in the first case (site Zn-CHC) and [Sγ (Cys(58)), Sγ (Cys(67)), Nϵ(His(73))] in the second (site Zn-CCH) from the initial values down to 2.5 Å in 10 ps. The final configurations of sites Zn-CHC (segment S1: 15–23) and Zn-CCH (segment S2: 58–73) are displayed in Figure 3, left and right panels, respectively. All calculations were performed with the LAMMPS code (Plimpton, 1995).

We interpret the fact that we have been able to get Zn-CHC and Zn-CCH stable configurations already with very simple classical simulation techniques, as a strong indication of the high propensity to give raise to ZF domains as soon as the occurrence frequency of Cys and His along the protein (or in its environment) is sufficiently high.

### 2.1. Refining the Zn Binding Sites

In the previous section by means of empirical non-polarizable force fields we have built candidates Zn binding sites for the orf7a protein of SARS-CoV-2. A more realistic description of Zn–protein interactions, especially when Zn-S bonds occur, requires polarizable or reactive force fields (Zhang et al., 2012; Li and Merz, 2017). Semiempirical quantum mechanics methods, like those driven by the density-functional tight-binding approximated (DFTB) hamiltonian (Elstner et al., 1998), include polarization and charge transfer in the calculation of atomic forces and allow energy minimization of systems of thousands of atoms within periodic boundary conditions (Hourahine et al., 2020). In this subsection we explore the energy profile around the previously selected configurations, using a DFTB hamiltonian.

**Figure 2:**
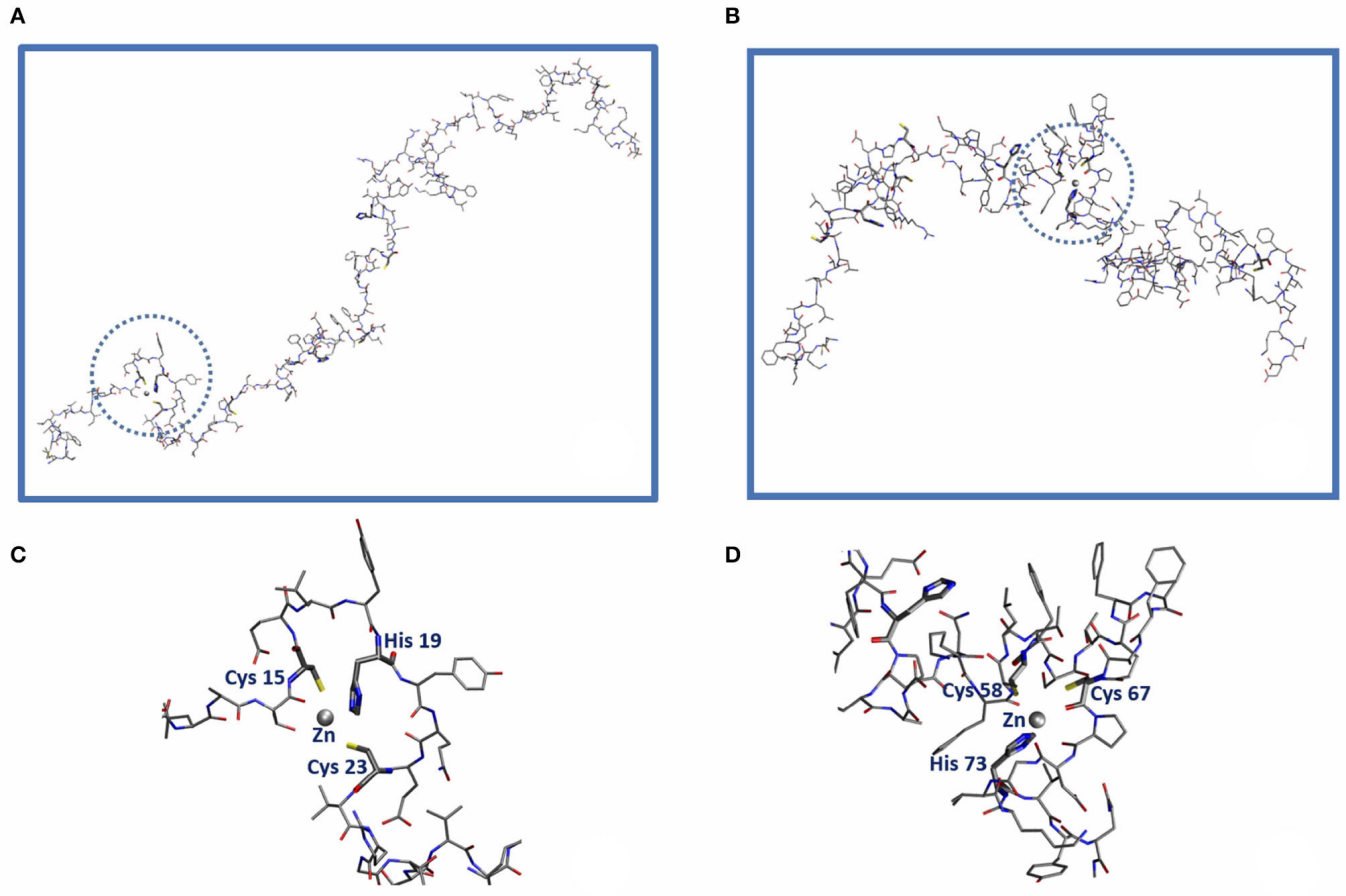
Sketch of the orf7a domains capable of hosting a Zn^2+^ ion (gray sphere) within the **C**ELY**H**YQE**C** 15-23 segment (Zn-CHC, left) and the **C**FSTQFAFA**C**PDGVK**H** 58-73 segment (Zn-CCH, right). The amino acid residues belonging to the first Zn coordination shell are highlighted in bold along the sequence. **(C,D)** Represent the blow-up of the local Zn^2+^ binding sites Cys(15)-His(19)-Cys(23) [Circled in **(A)**] and Cys(58)-Cys(67)-His(73) [circled in **(B)**], respectively.

Since the candidate Zn binding sites displayed in Figure 2 involve a limited protein region, we built model configurations of the systems of interest by truncating the protein around the ZF domains involved in Zn binding. We choose for the Cys(15)-His(19)-Cys(23) and Cys(58)-Cys(67)-His(73) binding sites the Ala_13_...Gly_26_ and the Asp_51_...Val_74_ segments, called for short S1 and S2, respectively, in the following. The N- and C-termini of the segments were capped with acetyl and N-methyl groups, respectively. Cys binding side-chains were assumed as deprotonated according to NMR measurements of ZF domains (Tochio et al., 2015).

For each one of the two segments we constructed a simulation box by inserting the corresponding amino acids together with Zn into an ortorhombic cell whose volume is minimized by rotating the protein segments. The cell was then filled of TIP3P water molecules (Jorgensen et al., 1983) with the help of the “solvate” utility of VMD (Humphrey et al., 1996). At this point the energy of the system was first minimized for as long as 2000 conjugate gradient (CG) steps by moving water molecules and capping groups only. The same force field as the one we used in the previous classical self-avoiding random walk simulation was employed here. The final configurations were then used as the initial configurations in the CG energy minimization performed with the DFTB hamiltonian. The DFTB+ code (Hourahine et al., 2020) was used with the znorg-0-1 parametrization (Moreira et al., 2009). The energy is assumed to be in a minimum when the maximal force component was lower than 0.01 Ha/Bohr. Convergence was achieved within 150 steps in all cases.

In order to estimate the formation energy of the Zn binding sites in the minimized configurations we obtained, we compare the former with two new systems that are obtained by exchanging the position of the Zn ion with that of the O of a water molecule lying far from the protein side-chains, upon transferring the entire water molecule where the Zn was located. After the exchange, the Cys side-chains turn out to be protonated. The exchange of Zn with a water molecule is represented by the chemical reaction

(1) $$\begin{array}{l} {\text{Zn}}_{\text{aq}}^{2+}+{\text{PH}2}^{\text{q}+2}\to {\text{ZnP}}^{\text{q}+2}+{2\text{H}}_{\text{aq}}^{+} \end{array}$$

where PH_2_ is the protein segment with protonated Cys side-chains (when not bound to Zn), ZnP represents the complex formed by Zn and the deprotonated protein.

The charge *q* of the protein is −3 and −2 for the S1 and S2 segments, respectively. The energy of each complex was therefore corrected for the Makov–Payne term (Makov and Payne, 1995), to take into account the difference in the cell net charge from the initial to the final state of the reaction (1).

The uncorrected energy of reactants is computed as the lowest energy of the system with Zn exchanged with a bulk water molecule. The energy of the product in reaction (1), ZnP, is that of the complex in the box. The energy of hydrated proton is, as usual, taken from experiments (Raffa et al., 2007). After correcting for the Makov–Payne term (Makov and Payne, 1995) one gets the numbers reported in Table 1. We stress that both numbers are negative, meaning that Zn is bound to the ZF domains of the segments S1 and S2.

**Table 1:** Energy variation (Δ*E*, kJ/mol) between the initial and final minimized configurations in the reaction (1) for our models of the binding sites S1: Cys(15)-His(19)-Cys(23) and S2: Cys(58)-Cys(67)-His(73).

**Segment**	**ΔE**
S1	−376
S2	−1,242

**Figure 3:**
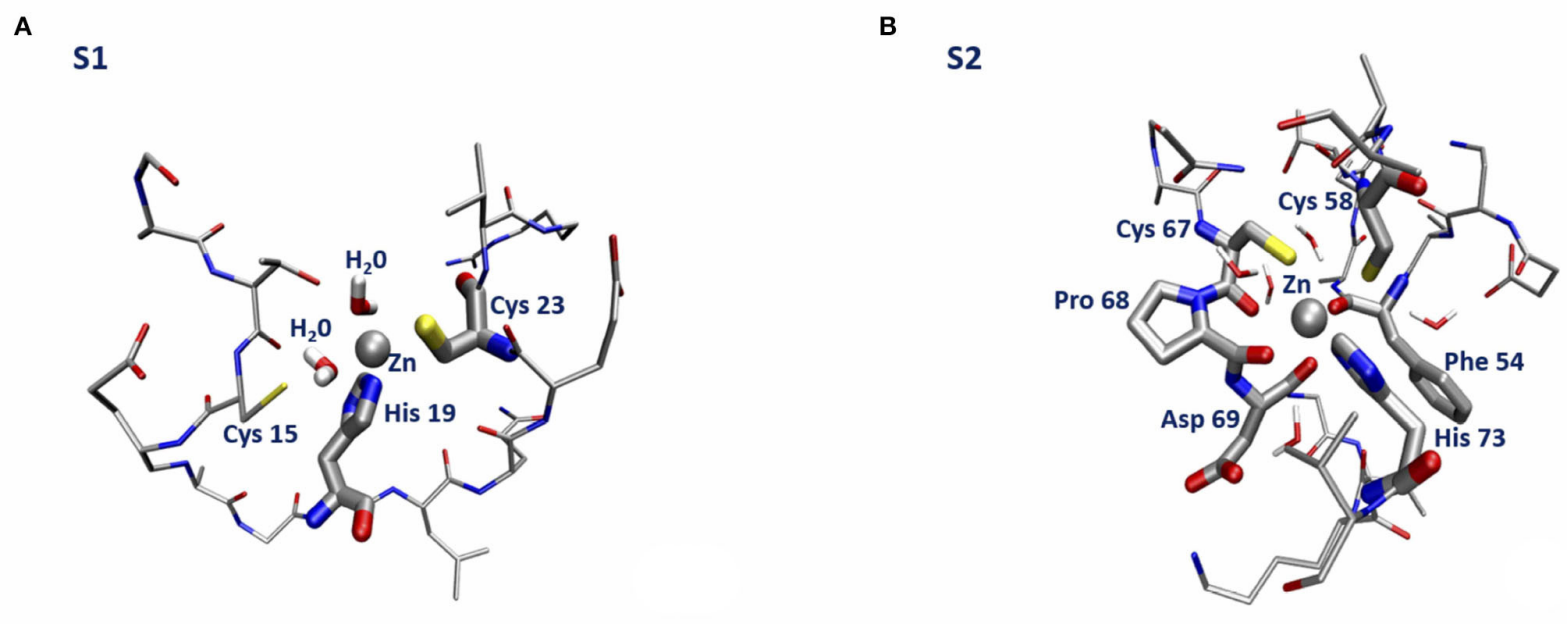
Sketch of the structure of the orf7a S1 **(A)** and S2 **(B)** segments at the end of the DFTB minimization.

The lowest energy configurations obtained in the DFTB approximation are displayed in Figures 3A,B. The coordination geometry around the Zn binding sites at the end of the DFTB minimization are reported in Tables 2, 3 where the distances of the atoms lying within 3 Å from the metal are reported. It is worth noticing that approximately the arrangement of the first shell Zn ligands is tetrahedral in the case of S1 and octahedral in the case of S2.

**Table 2:** Geometry of the first Zn coordination shell of the orf7a S2 segment at the end of the DFTB minimization.

**Residue**	**Atom**	**Distance [Å]**
Phe 54	O	2.30
Cys 58	S	2.78
Cys 67	S	2.38
Pro 68	O	2.32
Asp 69	O	2.23
His 73	N	2.00

*We report atoms lying within 3 Å from the metal*.

**Table 3:** Geometry of the first Zn coordination shell of the orf7a S1 segment at the end of the DFTB minimization.

**Residue**	**Atom**	**Distance [Å]**
His 19	N	1.97
Cys 23	S	2.29
Wat	O	1.99
Wat	O	2.04

*We report atoms lying within 3 Å from the metal*.

We end with a few remarks. First of all, we observe that the mechanical constraints acting within the bent protein regions are not sufficient to break the Zn bond. Secondly, we see that, while in the S1 Zn binding site Sγ (Cys 15) is replaced by a water molecule (see Figure 3A), the structure of the S2 Zn binding site is remarkably stable, as a consequence of the small probability for a water molecule to enter the first Zn coordination shell. The structural stability of the Zn bonds is ensured by presence of 2–3 carbonyl groups that keep Zn bound to the two Sγ atoms on the opposite side, with the His side-chain unperturbed by the protein small relaxation. Finally, we stress that the formation energy of the Zn bound complexes is dominated by the negative empirical contribution of the extraction of two protons into bulk water.

## 3. Discussion

With the help of classical MD simulations complemented with semiempirical quantum mechanics methods we have provided convincing evidence for the key role played by Zn^2+^ in stabilizing the orf7a (and possibly also) orf8 protein. Our working assumption is that orf7a (and/or orf8) can make Zn^2+^ available to BST2. The stability of Zn-S bonds, with S belonging to reduced sulfide groups, can in turn favor the breaking of some of the disulphide bridges of the folded BST2 tetherin, allowing the formation of inactivated orf7a/BST2 (and/or orf8/BST2) complexes with inhibition of the BST2 antiviral activity.

Based on the above considerations, we would like to argue that, although the affinity of Zn for the ZF domains is large (and larger than for other divalent cations of similar size available in cells, like Mg^2+^), one might think of displacing Zn^2+^ upon altering concentration in the host cells by temporary Zn deprivation and Mg augmentation. Indeed, situations have been identified in which, depending on the specific amino acids forming the metal coordination site, proteins can preferentially bind Mg^2+^ over Zn^2+^ (Gati et al., 2017).

Thus, cellular Zn deprivation, with Zn replacement by Mg and/or specific drugs based on Ag(I) or Au(I) (Kluska et al., 2020), might result in a significant slowing down of viral replication, owing to the inhibition of orf7a/BST2 and orf8/BST2 complexes formation. Circumstantial support for this hypothesis comes from the well-known, important role played by Zn in inflammation (Gammoh and Rink, 2017).

According to the scenario we have described, we are engaged in the long-term project of producing atomistic models of the orf7a/BST2 and orf8/BST2 systems, that can serve as reliable templates in the analysis of the forthcoming experiments aimed at unveiling the formation pathways and the detailed structure of these complexes.

The comparison to known related structures, i.e., the N-terminal ectodomain of orf7a of SARS-CoV-2 in the absence of Zn (PDB 6W37) and Ncp7 of HIV-1 in the presence of Zn (Morellet et al., 1998) (PDB 1ESK), will be the basis to model BST2 disulfide bond plasticity induced by modulation of orf7a/orf8 Zn binding.

The resulting predicted structures will also be useful for the forthcoming experimental studies aimed at validating (or disproving) the function of orf7a/orf8 proteins as diversion tools of the interferon-dependent antiviral response.

## Data Availability Statement

The raw data supporting the conclusions of this article will be made available by the authors, without undue reservation, to any qualified researcher.

## Author Contributions

All authors listed have made a substantial, direct and intellectual contribution to the work, and approved it for publication.

## Conflict of Interest

The authors declare that the research was conducted in the absence of any commercial or financial relationships that could be construed as a potential conflict of interest.
